# Optimal allocation of HIV resources among geographical regions

**DOI:** 10.1186/s12889-019-7681-5

**Published:** 2019-11-12

**Authors:** David J. Kedziora, Robyn M. Stuart, Jonathan Pearson, Alisher Latypov, Rhodri Dierst-Davies, Maksym Duda, Nata Avaliani, David P. Wilson, Cliff C. Kerr

**Affiliations:** 1 Burnet Institute, Melbourne, Australia; 2 Department of Epidemiology and Preventive Medicine, Monash University, Melbourne, Australia; 3 Complex Systems Group, School of Physics, University of Sydney, Sydney, Australia; 4 Department of Mathematical Sciences, University of Copenhagen, Copenhagen, Denmark; 5 Deloitte Consulting LLP, Arlington, USA; 6 Deloitte Consulting LLP, The USAID HIV Reform in Action Project, Kyiv, Ukraine; 7 Deloitte Consulting LLP, San Francisco, USA; 8 Institute for Disease Modeling, Seattle, USA

**Keywords:** Geographical, Optimization, Modeling, Resource allocation, Allocative efficiency, Ukraine

## Abstract

**Background:**

Health resources are limited, which means spending should be focused on the people, places and programs that matter most. Choosing the mix of programs to maximize a health outcome is termed allocative efficiency. Here, we extend the methodology of allocative efficiency to answer the question of how resources should be distributed among different geographic regions.

**Methods:**

We describe a novel geographical optimization algorithm, which has been implemented as an extension to the Optima HIV model. This algorithm identifies an optimal funding of services and programs across regions, such as multiple countries or multiple districts within a country. The algorithm consists of three steps: (1) calibrating the model to each region, (2) determining the optimal allocation for each region across a range of different budget levels, and (3) finding the budget level in each region that minimizes the outcome (such as reducing new HIV infections and/or HIV-related deaths), subject to the constraint of fixed total budget across all regions. As a case study, we applied this method to determine an illustrative allocation of HIV program funding across three representative oblasts (regions) in Ukraine (Mykolayiv, Poltava, and Zhytomyr) to minimize the number of new HIV infections.

**Results:**

Geographical optimization was found to identify solutions with better outcomes than would be possible by considering region-specific allocations alone. In the case of Ukraine, prior to optimization (i.e. with status quo spending), a total of 244,000 HIV-related disability-adjusted life years (DALYs) were estimated to occur from 2016 to 2030 across the three oblasts. With optimization within (but not between) oblasts, this was estimated to be reduced to 181,000. With geographical optimization (i.e., allowing reallocation of funds between oblasts), this was estimated to be further reduced to 173,000.

**Conclusions:**

With the increasing availability of region- and even facility-level data, geographical optimization is likely to play an increasingly important role in health economic decision making. Although the largest gains are typically due to reallocating resources to the most effective interventions, especially treatment, further gains can be achieved by optimally reallocating resources between regions. Finally, the methods described here are not restricted to geographical optimization, and can be applied to other problems where competing resources need to be allocated with constraints, such as between diseases.

## Background

To maximize impact, funding bodies must distribute limited resources in an optimal fashion, a process called allocative efficiency (AE). In recognition of this, the Joint United Nations Programme on HIV/AIDS (UNAIDS) Investment Framework for the Global HIV Response [1] has long encouraged national governments to invest in programs targeted to the populations at greatest risk of HIV infection. Mathematical models have proven useful in identifying the best way to go about this, with many models available for evaluating the impact of HIV-related interventions on population health outcomes [2–4]. Evidence from applications of these models suggests that considerable improvements could be achieved by reallocating funds across the mix of HIV interventions [5].

Although there are sophisticated tools available to assist countries in determining how to allocate funds between programmatic areas, there have only been a limited number of studies that have addressed the question of how to allocate funds for HIV responses between geographical regions. Examples include the question of how to optimally allocate antiretroviral services [6, 7] or healthcare clinics [8–10], as well as case-specific models that have been built to look at geographic prioritization of HIV responses in Kenya [11, 12] and sub-Saharan Africa [13]. For malaria, Walker et al. [14] developed a transmission model for all of Africa with 5 ×5 km “pixels” using interpolated data on rainfall seasonality, mosquito composition, and transmission intensity to define 55 unique “transmission settings”, with funding to meet specific reduction targets determined via simulated annealing. It was found to be less than half as much for pixel-level targeting compared to nationally uniform investments, illustrating the potential for large efficiency gains using this type of analysis. Thus, while these studies have shed a great deal of light on the magnitude of the gains that might be achieved via geographically-targeted responses, their specificity to particular countries and/or interventions limits the potential for widespread use by policymakers. Given the overwhelming evidence of geographical heterogeneity in HIV epidemics [15] and hence the emphasis placed by UNAIDS on the importance of geographical targeting [16], a more generalized tool to support geographical optimization of HIV responses would add considerable value to HIV response planning [17]. In particular, to date, there is no publicly available tool, adaptable to different national or subnational contexts, that is capable of calculating the distribution of funding between geographical areas that would result in the best HIV epidemic outcomes.

In this paper, we describe and discuss a novel algorithm for performing geographical analysis, which we have implemented for the Optima class of models. These models were originally developed for modeling HIV [18] and have subsequently been extended to other diseases. Optima models have been deployed in over 50 countries at the request of national governments seeking support in improving the allocative efficiency of their health responses [19–22]. The methods that we present here are completely generalized and can be used to determine the optimal distribution of funds across programmatic areas and geographical regions for HIV or any other disease, and do not depend on any specific features of the Optima model suite. The geographical optimization algorithm described here has been successfully used to inform HIV resource allocation in Côte d’Ivoire [23] and to optimize HIV spending across 44 countries comprising 80% of the global HIV burden [20].

**Fig. 1:**
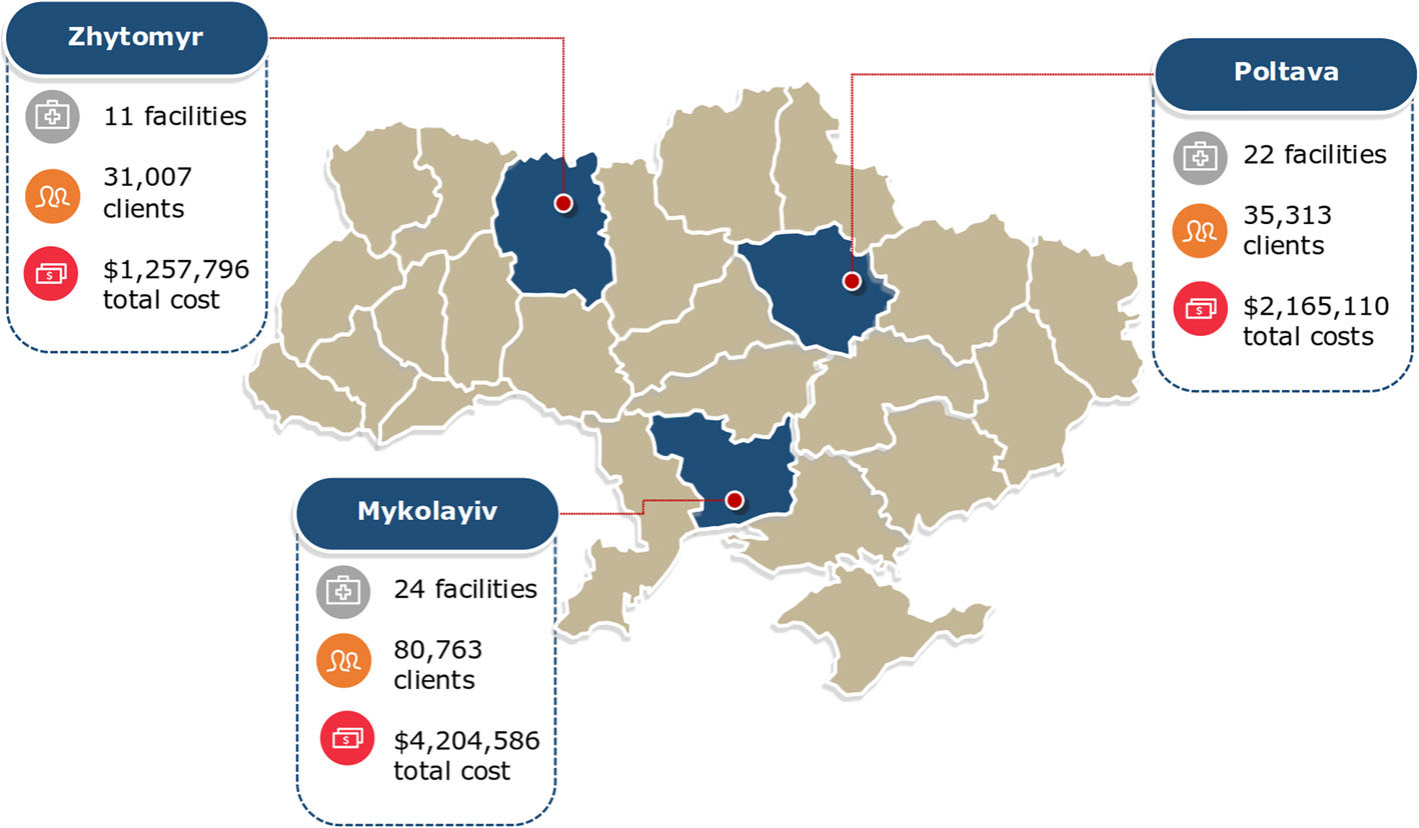
Oblasts of Ukraine used for the case study. Detailed epidemiological, expenditure, service, and delivery data were available for each oblast and were used to calibrate the Optima HIV model. These three oblasts (Zhytomyr, Poltava, and Mykolayiv) were chosen to represent low, medium, and high HIV prevalence regions, respectively. Map provided by and adapted with written permission from the USAID HIV Reform in Action Project [24]

To further illustrate the algorithm, we present the results of a computational study investigating how the optimal allocation for a single intervention (antiretroviral treatment) varies across regions that differ solely in terms of HIV prevalence. In addition, we present an example of applying geographical analysis via Optima HIV to three oblasts in Ukraine, under the auspices of the USAID HIV Reform in Action Project [24]. The oblasts used for the study are shown in Fig. 1. These particular oblasts – Zhytomyr, Poltava, and Mykolayiv – were chosen to allow for a comparison of costs and services across regions representing low (0.34%), medium (0.42%), and high (0.87%) HIV prevalences, respectively; hence, results from these three oblasts can be considered somewhat illustrative for the country as a whole [24]. Thus, this pilot study was conducted to determine what the general findings for optimization across oblasts would be and to estimate the potential gains from using this approach. In practice, funding would be reallocated on the national level and not only between these three oblasts. Furthermore, we emphasize that before a study such as this one could be translated into actionable national-level policy recommendations, it would be essential to perform additional data collection and complete a detailed exploration of supply-side and demand-side constraints.

## Methods

**Fig. 2:**
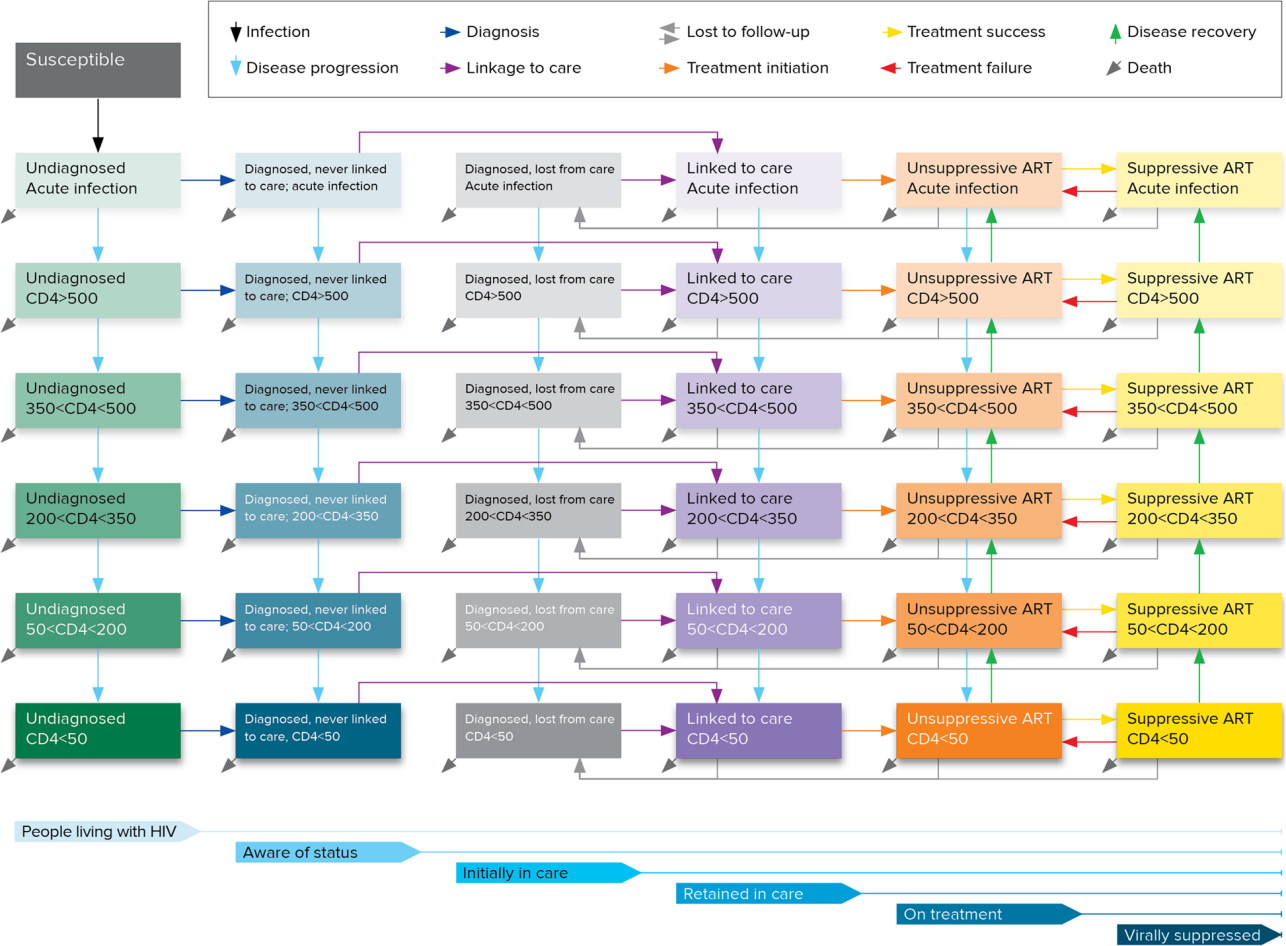
Compartmental structure of the Optima HIV epidemic model. This diagram shows the compartmental structure for a single population (e.g., females aged 25-34; the entire structure is duplicated for each population). Horizontal arrows represent movements between care states, while vertical arrows represent movements between health states

Each of the tools within the Optima suite is based on a compartmental model. Each model is defined by a network of *compartments*, corresponding to individuals subdivided among different subpopulations, disease states and care/treatment statuses. This can be considered an adaptation of the common susceptible-infected-recovered model structure [18, 25]. Figure 2 shows the compartmental model structure for Optima HIV. The rates at which people move between compartments (or enter, via birth or immigration, or leave, via death or emigration) depend on attributes that may include age, sex and other characteristics relevant to a disease. Interventions typically affect transition rates; e.g., a testing program affects the rate at which individuals move from undiagnosed to diagnosed states, while a treatment program may affect death and transmission rates.

There are three steps to an Optima analysis. First, the model is calibrated to match known epidemiological and behavioral data. For example, Optima HIV can be calibrated to HIV prevalence data points for subpopulations at specific time points and specific geographical locations, as well as new HIV infections, new HIV diagnoses, HIV-related deaths, and the number of people in each stage of the care cascade (i.e., diagnosed, linked to care, receiving treatment, and virally suppressed). For the Ukraine analysis presented here, data inputs, assumptions and calibrations were conducted in consultation with local HIV experts, including those from the USAID HIV Reform in Action Project and the National Public Health Center. The model was calibrated to each of the three study regions separately. Epidemiological inputs and model calibrations for each oblast are provided in Additional file 1, with further details on the consultation process and data sources provided in [24].

Second, Optima models incorporate nonlinear cost-coverage-outcome functions that relate the spending on HIV intervention programs to the coverage levels attained by these programs and the resulting behavioral outcomes. Thus, it is possible to define a package of interventions that associates current health expenditure with the present epidemic state, then examine how future projections change when funding is redistributed. Since interventions are implemented mechanistically and dynamically in the model, the impact and effectiveness of each intervention depends on both the state of the epidemic and the coverage levels of the other interventions. For example, the impact of a needle-syringe program will be reduced if there is high coverage of opiate substitution therapy (also called opiate agonist therapy); conversely, the impact of a testing program will be amplified if there is high enough funding for treatment programs so that newly diagnosed individuals can immediately begin treatment. Further detail on how cost-coverage and coverage-outcome functions are defined in Optima HIV is provided in [18] and [25]. Detailed expenditure and coverage data were available for each of the three oblasts in Ukraine. Data were collected from a two-tiered sampling approach to identify both regions (high, medium and low prevalence) and facilities offering at least one of nine HIV services available to residents. Regression-based extrapolation methods were used to extend per-patient unit costs and utilization to all facilities within each sampled region (Additional file 1: Table S1).

Third, the Optima models contain an optimization function that can be used to estimate the allocation of resources across programs that best addresses national targets whilst considering various logistic, political and ethical constraints. Further details are provided in [18, 25–27]. Briefly, Optima HIV uses the adaptive stochastic descent algorithm [28] to determine the allocation of funding across different programs that minimizes the objective being optimized, such as the number of new HIV infections, HIV-related deaths, and/or HIV-related DALYs.

### Defining regions

The key motivation for performing geographical optimization is that data are often available at the subnational level. Examples include surveys where responses are coded by location, such as the Demographic and Health Surveys (DHS) and AIDS Indicator Surveys (AIS) [29, 30], Population-based HIV Impact Assessment (PHIA) [31], and prevalence estimates made available via antenatal and sexual health clinics [32]. In some cases even continuous data are available, such as vegetation coverage (which can be used as a proxy for urban versus rural areas), although such data are typically more relevant for diseases with a strong environmental component such as malaria [14]. Where data are not directly available, spatial statistics methods exist in order to extrapolate between discrete data points and thus cover the full area of interest [5, 33–35]. For example, DHS surveys are conducted at individual sites, but their findings can be interpolated to other locations that were not directly measured.

The simplest way to include geographical divisions in an Optima model is to add additional populations corresponding to the different regions, modifying their properties where necessary to reflect data differences between locations. In this way, geographical location is considered a population attribute like age, sex, and so on.

However, there are significant practical limitations to this approach. The run time for a single iteration of the model is proportional to the number of populations, while the number of iterations required for optimization is roughly proportional to the square of the number of programs. Thus, performing a geographical analysis by duplicating the set of population groups and programs for each of the *N* regions means that the analysis is likely to take *N*^3^ times longer to run. While potentially feasible for 2 or 3 regions, this approach quickly becomes computationally impractical for even a modest number of regions. Thus, the simplification is made to treat each region as being independent. This can be used to simulate a large number of regions, including at the international level [20].

Ideally, a user would perform geographical analysis by combining a number of existing and calibrated models into a “portfolio”. For example, this is typically the approach taken to estimate optimal resource allocations among a number of countries. However, at the subnational level, sufficient data to inform individual models often do not exist. For example, while the net population sizes and prevalences of both a country and its component regions may be well known, the split of a key population across these regions can be more difficult to determine, especially in cases where these populations are marginalized.

To cater for situations where little or no information is available about the distribution of populations across regions, as for example can be the case for key populations in concentrated epidemics [36], we developed an automated algorithm for subdividing a single national-level model into a number of region-level models. This method partitions subpopulations among regions proportionally, i.e.

1 $$  s_{ri} = \frac{s_{i} s_{r}}{s},  $$

where *s*_*ri*_ is the size of subpopulation *i* in region *r*, $s_{i}=\sum _{r} s_{ri}$ is the corresponding national-level size of subpopulation *i*, $s_{r}=\sum _{i} s_{ri}$ is the total population size for region *r*, and $s=\sum _{r}\sum _{i} s_{ri}$ is the total national population size. For example, if there are 10,000 people who inject drugs (PWID) in a country of 1,000,000 people, and this country has 300,000 people in region A, 500,000 in region B, and 200,000 in region C, then we assume the PWID population is split by the ratio 3,000:5,000:2,000 across the three regions. Of course, the terms “region” and “country” can be replaced as appropriate for the level the partitioning is applied at.

Disease prevalence is more complex and cannot be scaled linearly. For example, if the national prevalence of a particular disease is 40% and prevalence within a particular region is 80%, then a national prevalence of 60% within a particular subpopulation could naively be scaled to an invalid 120% value at the region level. To avoid this issue, our automatic subdivision method defines population prevalence in each region as

2 $$\begin{array}{*{20}l}  p_{ri} &= \frac{R}{R-1+1/p_{i}}, \end{array} $$

3 $$\begin{array}{*{20}l} R &= \frac{p_{r}\left(1-p \right)}{p\left(1-p_{r} \right)}, \end{array} $$

where *p*_*ri*_ is the prevalence for population *i* in region *r*, $p_{i}=\sum _{r} \left (p_{ri} s_{ri} \right)/s_{i}$ is the corresponding national-level prevalence for population *i*, $p_{r}=\sum _{i} \left (p_{ri} s_{ri} \right)/{s_{r}}$ is the total prevalence in region *r*, and $p=\sum _{r}\sum _{i} \left (p_{ri} s_{ri} \right)/s$ is the total national prevalence. Note that these equations require *p*_*i*_ (national-level prevalence for each subpopulation) and *p*_*r*_ (overall prevalence for each region) as input data.

The automatic subdivision method represented in Equations (1) and (2) need only be used if specific data on region-level subpopulation sizes and prevalences are not available. In addition, model recalibration is typically necessary if automatic subdivision is used. Of course, the fewer region-specific data are used, the less informative the geographical analysis will be: ideally there would be region-specific data on epidemiological and behavioral indicators for each subpopulation as well as region-specific data on program costs, impacts, and constraints.

### Budget-outcome curves

Once the model for each region has been defined, each one can be run and optimized independently from the others. This allows the marginal impact of shifting funding between regions to be determined, via a function called a *budget-outcome curve* (BOC).

A BOC describes the relationship between a given amount of funding in a region and the best possible “outcome” that can be attained for that amount of funding in that region. The outcome is user-defined and is typically the number of new infections, deaths, or disability-adjusted life years (DALYs) incurred over a specified time period; the outcome can also be a weighted sum of two or more of these.

Figure 3 demonstrates how a BOC is constructed by illustrating the process that was performed for Mykolayiv. The algorithm proceeds as follows:

1. The baseline budget (i.e., the current allocation of funding to each program) is optimized using Optima’s built-in optimization algorithm to determine a starting point for the BOC. By definition, the optimal budget has a better (or, at worst, equal) outcome compared to the baseline (top right panel).

**Fig. 3:**
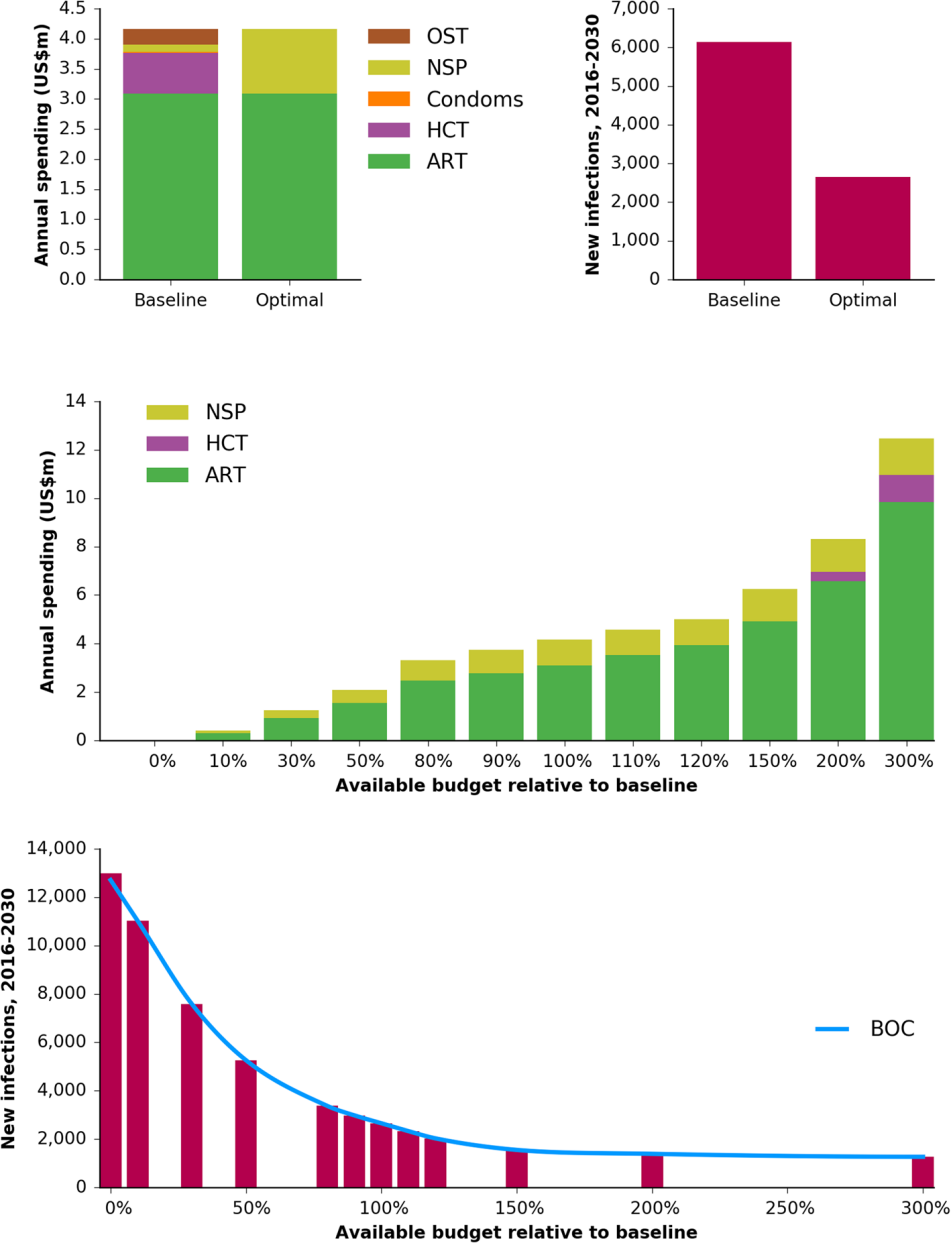
Construction of the BOC for Mykolayiv. The first step is to optimize the baseline budget (top left), which improves the outcome (top right; here using the example of minimizing the number of new HIV infections). The total budget is then scaled up and down and re-optimized (middle), resulting in a “staircase” of outcomes that can then be interpolated, thereby forming the BOC, with spending on the *x*-axis and the outcome (new infections) on the *y*-axis (bottom). Abbreviations: ART, antiretroviral therapy; HCT, HIV counseling and testing; NSP, needle-syringe program; OST, opiate substitution therapy; BOC, budget-outcome curve
2. The total budget is scaled both up and down, and optimization is performed for each new budget, using the optimized original budget (labeled “100%”) as a starting point. This step is known as constructing an optimal expansion pathway or investment staircase [37]. In many cases the budget allocations are simply scaled versions of each other (e.g., the 150% budget allocation is almost exactly the 120% budget allocation scaled by a factor of 1.25), but there are also points at which the mix of programs changes (e.g., the 200% budget allocation includes funding for HCT, while the 150% budget allocation does not). Since real-world funding shifts are often relatively small, it is especially important to sample points around the current funding level. Because each point on the BOC requires a budget optimization to be run, and since optimizations are computationally expensive, BOCs are typically calculated based on no more than 10–20 points (including zero spending and a very large amount approximating unlimited spending).
3. The budget-outcome points determined in step 2 are interpolated to create a continuous function. This interpolation is typically done using piecewise cubic Hermite interpolating polynomials (“PCHIP”), a type of spline created by fitting smooth polynomials to the data [38].

**Fig. 4:**
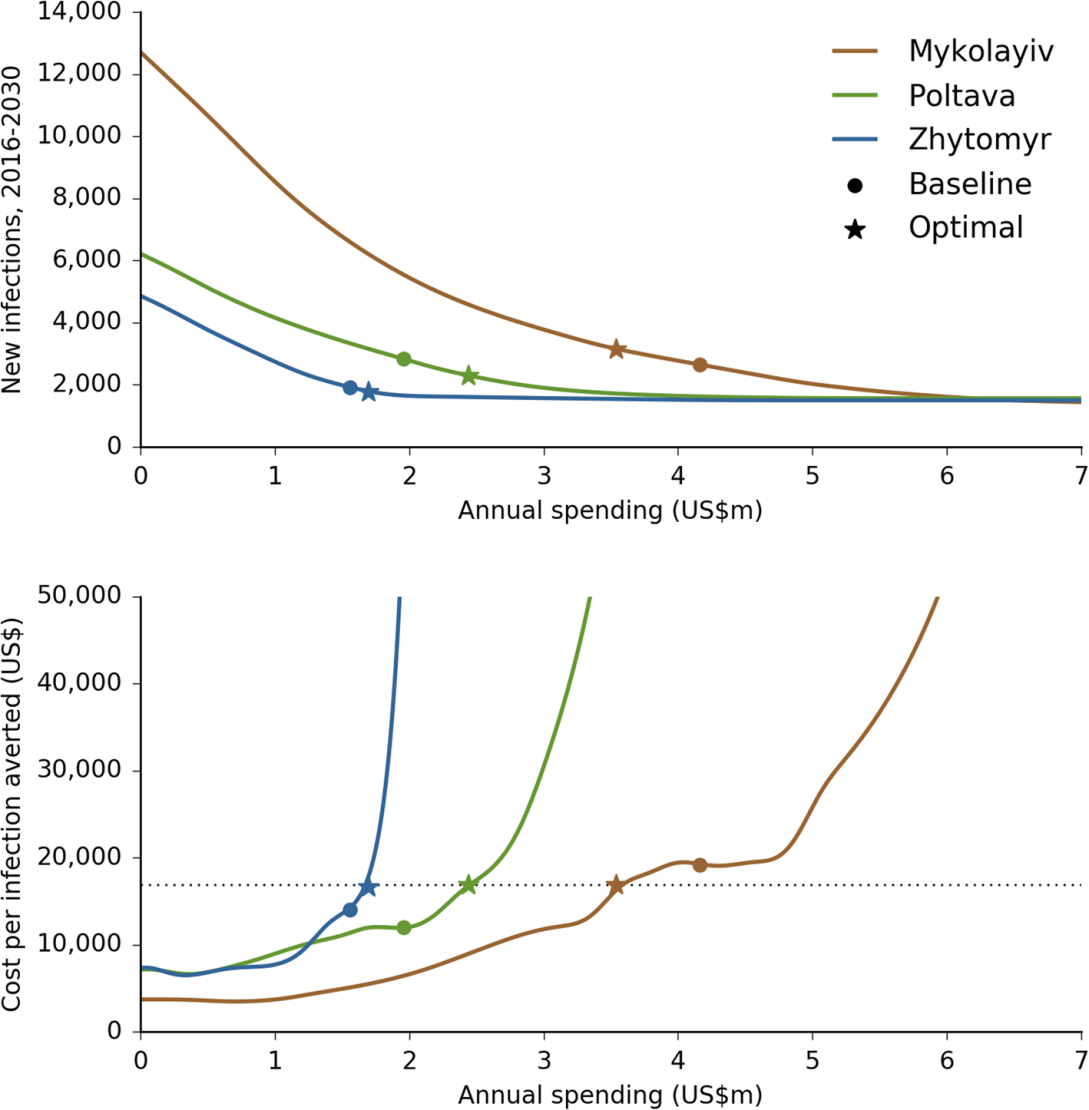
Example budget-outcome curves (BOCs). The top panel shows the BOC for each oblast, including the optimally-allocated annual baseline spending amount (circle) and the geographically-optimized annual spending amount (star) for the outcome of minimizing new HIV infections. The bottom panel shows the estimated cost per infection averted, which is the inverse of the negative first derivative of the BOC and effectively equivalent to a type of incremental cost-effectiveness ratio. Note that, at the optimum, this quantity is equal across the three BOCs

Several example BOCs are shown in Fig. 4. Formally, a BOC can be defined as the function *O*_*r*_(*b*_*r*_) for region *r*, where the estimate of the outcome *O*_*r*_ for any given input budget *b*_*r*_ is achieved via spline interpolation.

Since a BOC describes the relationship between budget and outcome, it is closely related to the definition of an incremental cost-effectiveness ratio (ICER). In particular, the negative of the first derivative of a BOC is the incremental change in outcome for an incremental change in budget, and is thus the reciprocal of an ICER. The usual definition of the ICER is used to summarize the cost-effectiveness of a single health care intervention by comparing the outcomes obtained under two alternatives (implementing vs. not implementing the intervention) with the cost of implementation [39, 40]. By contrast, this variant summarizes the cost-effectiveness of an incremental change in the entire response in a given region *r*, and compares the outcomes of two alternatives: allocating resources of *b*_*r*_ to region *r* vs. allocating resources of *b*_*r*_+*Δb*_*r*_ (where *Δb*_*r*_ represents a small increase in the budget to region *r*).

### Optimizing across regions

**Fig. 5:**
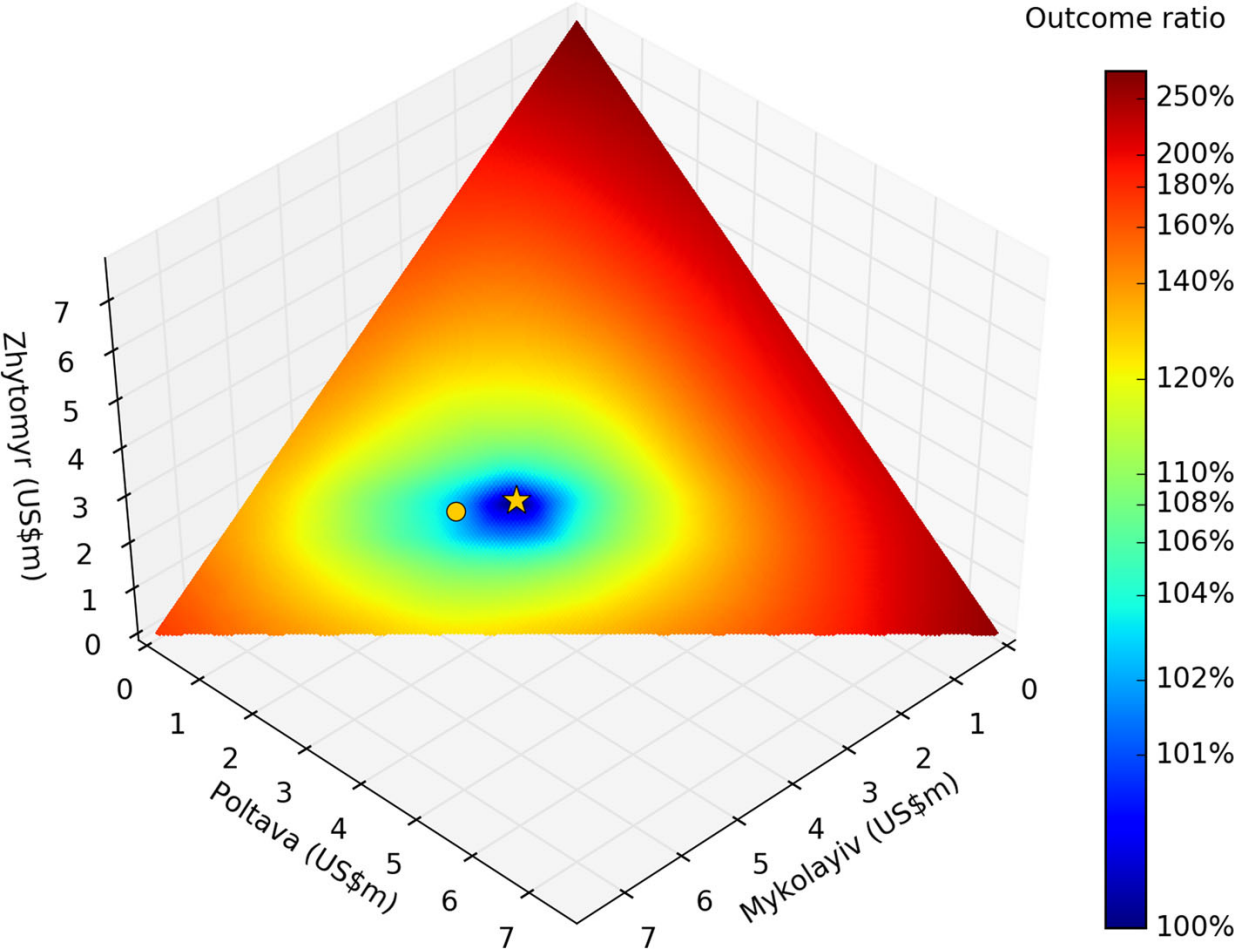
Solution-space hyperplane. Each axis shows the annual spending for each oblast, such that the total spending remains constant; the logarithmic color scale shows the ratio of the outcome for each possible inter-region allocation (here, the cumulative number of new HIV infections from 2016–2030), compared to the optimal inter-region allocation. In particular, outcomes are shown for the baseline allocation (circle) and the geographically-optimized allocation (star), indicating that the baseline inter-region allocation is already very close to optimal

The final step of geographical analysis is to decide how to optimally distribute a fixed budget *B* across the regions. If *b*_*r*_ is the budget for region *r* and *O*_*r*_(*b*_*r*_) is the outcome in region *r* given budget *b*_*r*_, then this problem is equivalent to minimizing net outcome $O=\sum _{r} O_{r}(b_{r})$, subject to the constraint that $\sum _{r} b_{r} =B$. Without the constraint that the total budget *B* remains constant, for *n* regions, possible allocations lie in an *n*-dimensional hyperspace (e.g., if there are 10 programs in the budget, the space of all possible allocations will be a 10-dimensional cube), and the solution with the best outcome would simply be to have maximum funding in all regions. However, with the constraint of fixed total budget, solutions are restricted to lying on an (*n*−1)-dimensional hyperplane. For example, the 2D hyperplane corresponding to the 3D solution space for Ukraine is shown in Fig. 5.

Although this looks like a relatively straightforward minimization problem, dangers still lurk. The naïve expectation is that a geographically optimal distribution of funds is equivalent to finding the point where the derivatives of all BOCs are equal (as illustrated in Fig. 4), i.e.:

4 $$ \frac{d O_{1}(b_{1})}{d b_{1}} = \frac{d O_{2}(b_{2})}{d b_{2}} =... = \frac{d O_{N}(b_{N})}{d b_{N}}.  $$

At this point, incrementally shifting any funding from one region to another would have zero change in net outcome. However, this condition is neither necessary nor sufficient to ensure the global optimum. It is not necessary because there may be regions *x* and *y* such that $\left (d O_{x}(b_{x}) / d b_{x} \right) > \left (d O_{y}(b_{y}) / d b_{y} \right)$ for all *b*_*x*_ and *b*_*y*_ (so the equality never holds), and it is not sufficient because there may be multiple points that satisfy this condition. This is because although BOCs are almost always monotonic themselves (i.e., always decreasing), their derivatives are often not. For example, in Fig. 4, the cost per infection averted in Mykolayiv is approximately US$19,950 for annual spending amounts of both US$4.0m and US$4.5m, meaning that there is not necessarily a unique solution for the point at which the cost per infection averted is the same in each region.

To solve this, we employ a type of greedy grid search algorithm. The principle of the algorithm is to start with no funding allocated to any region and then to progressively allocate funding to the region where it has the most impact until the entire available budget *B* has been allocated. More precisely, the algorithm works as follows:

1. Begin by defining a region array **r**=[1,2,…,*N*], where *N* is the number of regions. Next, initialize the budget for each region to $0, i.e., define an array $\mathbf {b}^{0} = \left [ b^{0}_{1}, b^{0}_{2}, \ldots, b^{0}_{N} \right ] = [0,0,\ldots,0]$. Here, the superscript 0 represents the number of iterations of the algorithm; thus $b_{r}^{j}$ refers to the budget allocated to region *r* at the *j*-th iteration of the algorithm.
2. Choose a number *K* of trial budgets and a corresponding array, **k**=[1,2,...*K*]. Next, create an (initially) identical array of trial budget values for each region, $\mathbf {x}_{r}=\left [x_{r}^{k}\right ]_{k=1}^{K}$, from $0 up to the maximum total budget *B*, where
  5 $$  x_{r}^{k} = \exp\left(\frac{\log\left(B\frac{k}{K}\right) + \log(B)\frac{k}{K}}{2}\right).  $$
  The spacing of points defined by Equation 5 is used in order to place equal emphasis on absolute funding amounts (the linearly-spaced first term of the equation, which ensures sufficient sampling for large budgets) and relative funding amounts (the exponentially-spaced second term of the equation, which ensures sufficient sampling for small budgets). For example a choice of *K*=2000 and a total budget of $10 million roughly corresponds to average increments of $5000, with an initial increment of $100 and a final increment of $40,000.
3. For each region *r*, use the corresponding budget-outcome curve *O*_*r*_ to evaluate the outcome (e.g., number of new HIV infections) for every trial budget value $x_{r}^{k}$, i.e. $O_{r}(x_{r}^{k})$. This step generates *N*×*K* points, which is the solution space over which the grid search is performed.
4. For each iteration *j* of the algorithm, perform the following steps:
  - For the set of trial budgets for each region, **x**_*r*_, calculate the trial budget increments, $i_{r}^{k} = x_{r}^{k} - b_{r}^{j}$, and exclude any trial budgets for which (i) funding for a region would remain the same or decrease (i.e., $i_{r}^{k} \le 0$), or (ii) accepting the increment would exceed the total available budget (i.e., $\sum _{r=1}^{N} b^{j}_{r} + i_{r}^{k} > B$). If there are no trial budgets remaining after this step, terminate the loop.
  - For each remaining trial budget for each region, $x_{r}^{k} \in \mathbf {x}_{r}$, calculate the marginal cost-effectiveness $M(x_{r}^{k})$:
    6 $$ M(x_{r}^{k}) = \frac{O_{r}(b_{r}^{j}) - O_{r}(x_{r}^{k})}{ x_{r}^{k} - b_{r}^{j}}.  $$
    Since funding for a region cannot decrease (i.e., $x_{r}^{k} - b_{r}^{j} > 0$), and, in the real world, more funding cannot lead to a worse outcome (i.e., $O_{r}(b_{r}^{j}) \ge O_{r}(x_{r}^{k})$), it follows that marginal cost-effectiveness cannot be negative (i.e., $M(x_{r}^{k}) \ge 0$).
  - Select the region *r*^∗^ and funding level *k*^∗^ that has the greatest marginal cost-effectiveness, i.e., where $M(x_{r^{*}}^{k^{*}}) = \max \left (M(x_{r}^{k})\right)$ for all regions *r* and remaining budget levels *k*. (In the case of a tie, i.e. if two or more regions have the same marginal cost-effectiveness, allocate funding to the region with the least funding.)
  - Allocate the budget $x_{r^{*}}^{k^{*}}$ to region *r*^∗^ (i.e. set $b_{r^{*}}^{j+1} = x_{r*}^{k*}$).
5. If necessary (i.e., if the allocated budget does not exactly match the total budget *B* due to the discrete steps of **x**), rescale all regional budgets *b*_*r*_ so their sum equals the total budget, i.e. $b_{r}^{'} = b_{r} \frac {B}{\sum _{r=1}^{N} b_{r}}$.

Despite the large number of individual calculations in this algorithm, since it does not require the model itself to be rerun, it is quite computationally efficient. For example, in the Ukraine case study (*N*=3 and *K*=2000), this algorithm takes several seconds to run on a standard laptop, compared with roughly an hour to compute the three BOCs.

### Implementation in Optima HIV

**Fig. 6:**
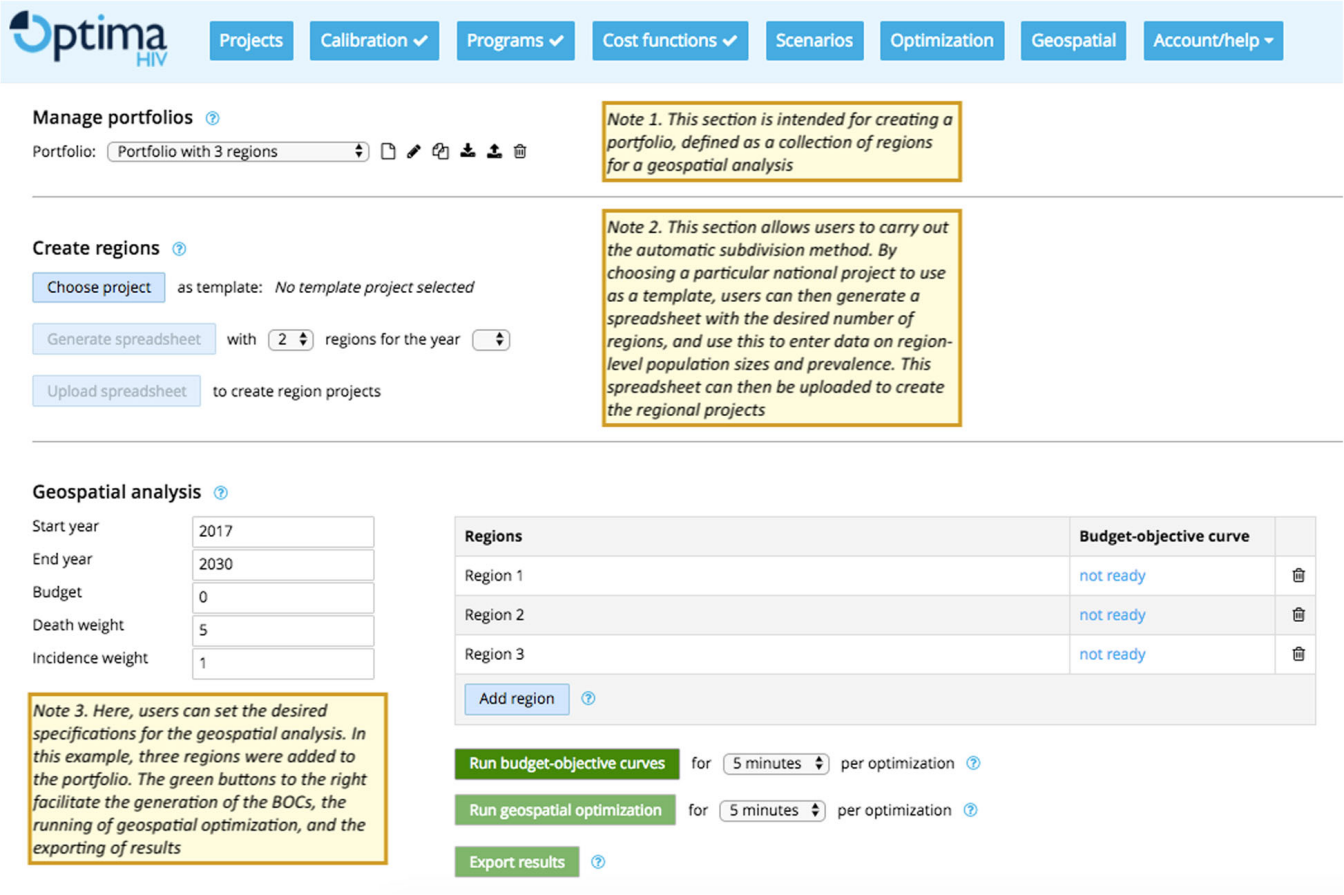
Interface for running geographical optimizations. Yellow notes indicate the purpose of each section of the interface: the first section of the geographical analysis interface allows users to create a portfolio; the second section allows them to define regions, and the final section allows them to generate BOCs, run geographical optimization, and export the results. Image adapted from the public domain Optima HIV webapp, http://hiv.ocds.co

The geographical analysis methods described here have been implemented in the standard Optima HIV software distribution. Optima HIV is an open-source, public domain software tool that is made freely available for both users (via the webapp, http://hiv.ocds.co) and developers (via GitHub, http://github.com/optimamodel/optima). Instructions for use are available at ocds.co/user-guide. Figure 6 demonstrates the connection between the steps outlined above and their implementation in the Optima HIV software.

## Results

Here we present results from applying the algorithm described above to two cases: a computational analysis of synthetic data corresponding to regions of smoothly varying prevalence, and the previously discussed case study covering three oblasts in Ukraine.

### Computational analysis

This section describes an exploration of the geographical algorithm using synthetic data. For this example, we created a simple epidemic model of 1.8 million people, split approximately equally between general population males and females aged 15-49. HIV prevalence in 2018 was set to 1.5% in males and 2.0% in females, for a total of approximately 34,000 PLHIV. HIV transmission was assumed to occur solely through heterosexual partnerships, with an assumed 100 acts per year with 14% condom use; condoms were assumed to be 95% effective and per-act transmission rates without condoms were assumed to be 0.08% for females (i.e., receptive acts) and 0.04% for males (i.e., insertive acts), corresponding to an average annual probability of infection for a serodiscordant partnership of approximately 6.9% for females and 3.5% for males. The only interventions in the model were (1) HIV testing and counseling (including linkage to care), representing 6% of people being tested per year, and (2) antiretroviral therapy (including treatment initiation and adherence), covering 3,200 people (corresponding to coverage of 10%).

**Fig. 7:**
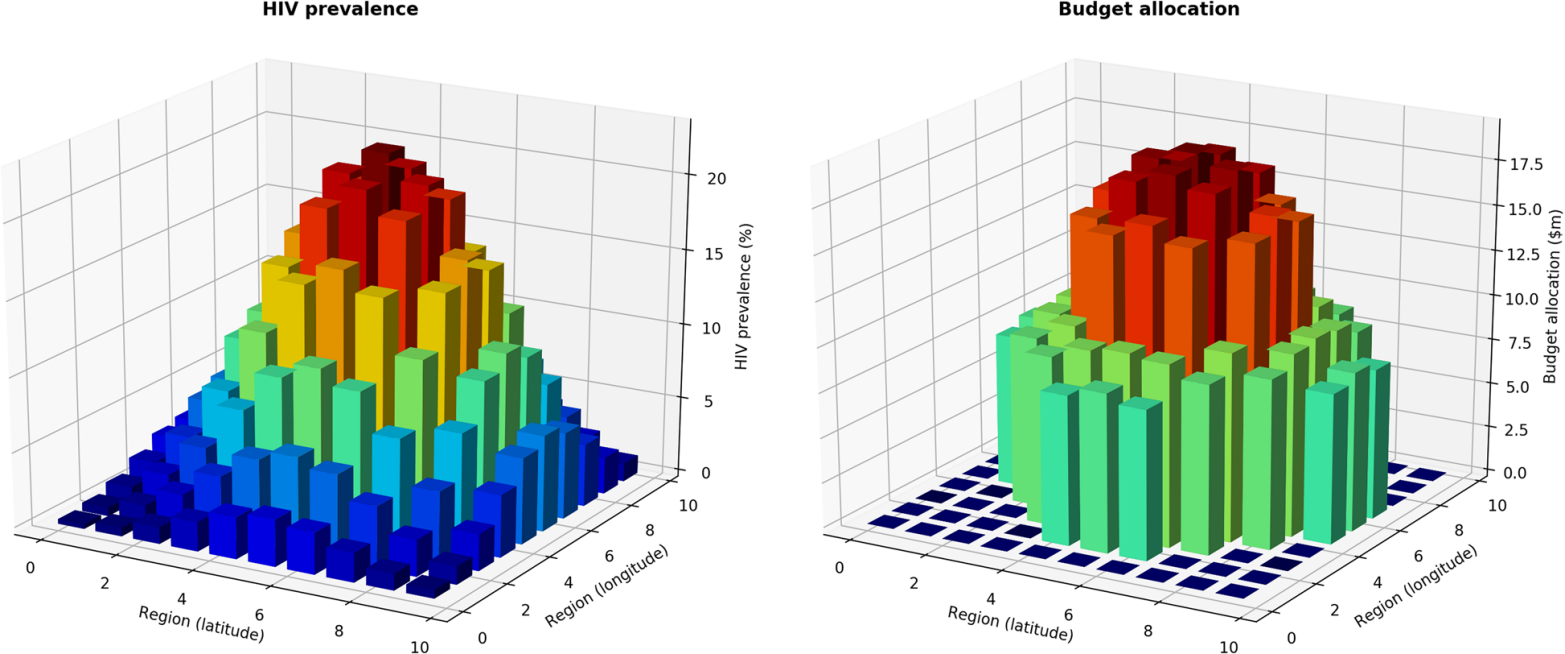
Nonlinearities in a hypothetical example of geographical optimization. Initial HIV prevalence (left) and the resultant optimal budget allocation (right) for 100 contiguous regions

From this “default” model, 100 separate regions were created in a 10 ×10 grid. All regions were identical to the model described above, except that the number of acts was modulated by a factor of 5 from the edges to the center (i.e., from 50 to 250 acts per year). This created a nearly 100-fold difference in prevalence by 2030: from 0.3% in the corners to 23% in the center (Fig. 7A). Initial funding was set to be equal across all regions at US$7m.

HIV testing was held constant and geographical optimization was applied to the funding of ART across the 100 regions. As shown in Fig. 7B, funding for ART did not scale linearly with prevalence. Although (final) prevalence varied smoothly from 0.3% to 23%, funding instead showed three distinct levels: the 40 lowest-prevalence regions received zero funding; another 28 regions received moderate funding (roughly equal to the initial funding value of US$7m), while the 32 highest prevalence regions received roughly double their initial funding. These nonlinearities result from positive feedback effects within the population, since the impact of treatment (especially treatment-as-prevention) depends on both the initial differences between each region as well as the different dynamics that result from these initial differences. The reallocation of funds reduced new infections and deaths by 5.7% compared to uniform funding. This example illustrates that even in a simple situation, the optimal geographical distribution of funding is not necessarily apparent from epidemiological data alone.

### Application to Ukraine

This section describes the application of geographical analysis to the three Ukrainian oblasts of Mykolayiv, Poltava and Zhytomyr. Each oblast included 11 subpopulations: female sex workers (FSW); clients of female sex workers; men who have sex with men (MSM); people who inject drugs (PWID); prisoners; and general population males and females aged 0–14, 15–49, and 50+ years. Each oblast also included five interventions: antiretroviral therapy (ART, which corresponds to treatment rates), HIV counseling and testing (HCT, which increases diagnosis rates), condom programs (which increase condom usage in casual partnerships), needle-syringe programs (NSP, which reduce needle sharing rates among PWID), and opiate substitution therapy (OST, which reduces injections among PWID). All programs were implemented in each of the oblasts, although coverage was allowed to be reduced to 0%, except for ART, which was not allowed to decrease as a proportion of the total budget. Further details are provided in Additional file 1.

The results presented here include constraints on the algorithm to reflect logistical, ethical, and political considerations, namely: once people begin ART, they cannot be removed; programs cannot exceed maximum coverage levels, typically 85-95% depending on the program and target population group; and, as described below, in one analysis, funding to testing was not allowed to decrease. Thus, the BOCs which the optimizations shown here are based on do not necessarily represent the mathematically optimal solutions. (In contrast, the BOCs shown in Fig. 4 are mathematically optimal, for the special case of minimizing new infections only.) Here, optimizations were performed to minimize DALYs. DALYs were calculated using the “hybrid approach” [41], i.e. the sum of years lived with disability (i.e., the number of people in each disease state multiplied by the disutility of that disease state) and sum of years of life lost (i.e., life expectancy minus age at death) [42].

**Fig. 8:**
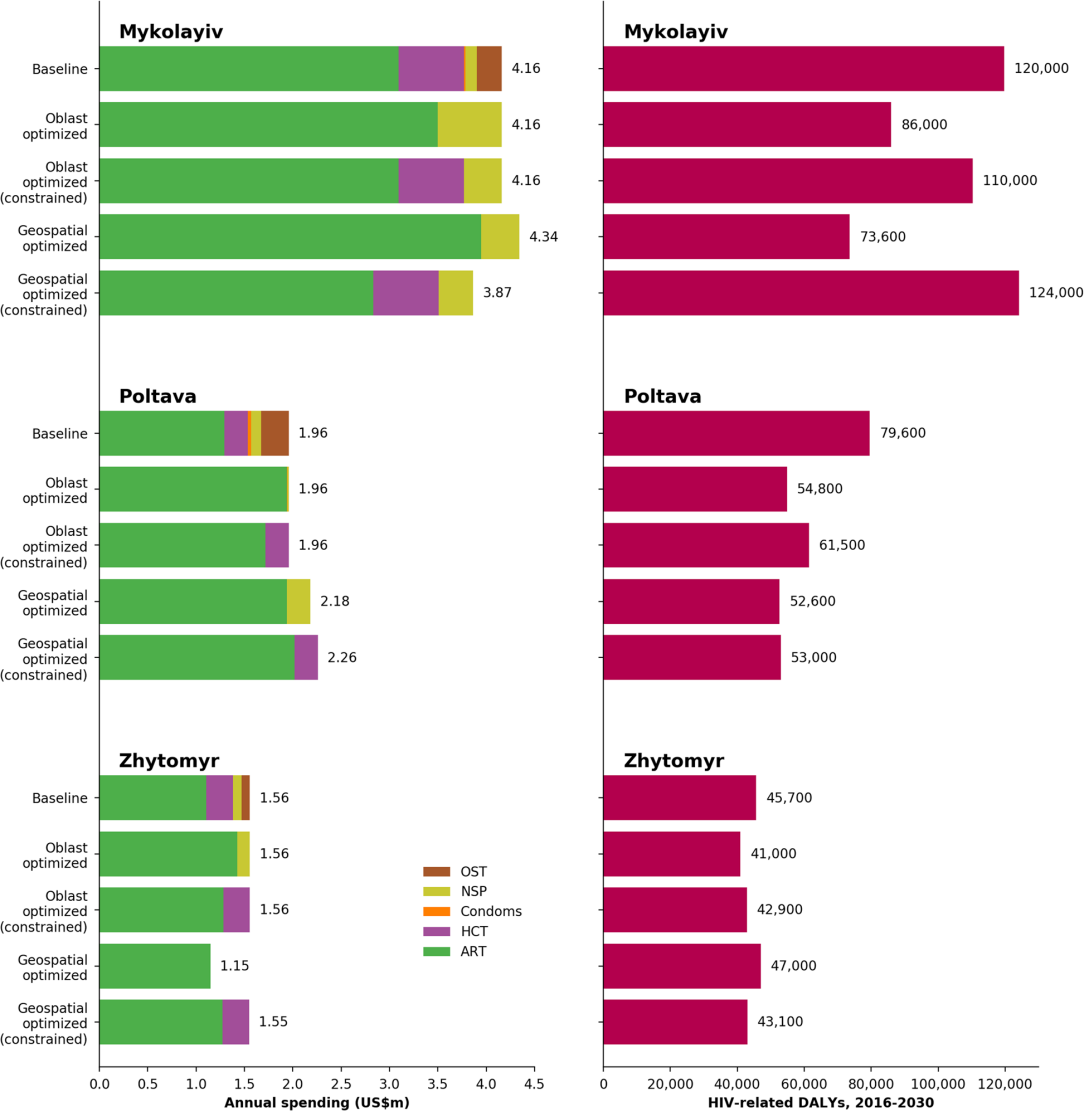
Baseline and optimal budget allocations with corresponding outcomes for each oblast. Optimal budget allocations are shown for both intra-oblast optimization (where the budget for that oblast is the same as baseline) and for geographical optimization (where funding is allowed to shift between oblasts)

Figure 8 shows the budget allocations and corresponding outcomes for each oblast given baseline spending, intra-oblast optimized spending, and inter-oblast (geographically) optimized spending, as well as results with spending on HIV testing constrained, due to in-country political constraints. Intra-oblast optimization results in a more targeted response, focusing on just two key programs in the unconstrained case (antiretroviral therapy and needle-syringe programs). The intuitive explanation for this result is that ART and NSP are the most cost-effective programs for reducing HIV-related deaths and new HIV infections, respectively, and these are the two main contributors to DALYs. Shortening the 15-year timeframe of the analysis would shift funding towards treatment; conversely, lengthening the timeframe would shift funding towards prevention (specifically, needle-syringe programs).

In each case, the unconstrained optimization led to many more DALYs averted than the constrained optimization (24,000, 6,000, and 2,000 additional DALYs averted for Mykolayiv, Poltava, and Zhytomyr, respectively). Note that although treatment and needle-syringe programs have the highest impact in terms of averting new HIV infections and HIV-related deaths, funding other programs, especially opiate substitution therapy, is highly desirable due to the many other benefits that these programs may have in addition to averting HIV infections. This indicates the need for increased resources to fund a sustainable and multi-purpose response. The defunding of the HIV counseling and testing program in the unconstrained case was due to the fact that in each oblast, there were sufficient numbers of PLHIV who were diagnosed but not receiving treatment such that ART could be scaled up without any additional investment in testing.

Geographical (inter-oblast) optimization shifts resources away from the oblast with lowest HIV prevalence (Zhytomyr) and towards the oblasts with higher HIV prevalence (Poltava and Mykolayiv). As shown in the right panel, intra-oblast optimization reduces the total number of DALYs across all oblasts; compared to intra-oblast optimization, geographical optimization results in further moderate improvement in Mykolayiv and a small improvement in Poltava, at the expense of more DALYs in Zhytomyr. According to the optimization analysis, allocation shifts both within and between oblasts were relatively small, indicating that Ukraine’s HIV response in these three oblasts is already relatively close to optimal.

Oblast-level optimization produces large forecasted reductions in DALYs over 2016-2030 in Mykolayiv (from 120,000 to 86,000), with smaller but still notable changes in Poltava (from 80,000 to 55,000) and Zhytomyr (from 46,000 to 41,000). Geographical optimization increases DALYs in Zhytomyr (to 47,000), but reduces them to 74,000 and 53,000 in Mykolayiv and Poltava, respectively. Thus, intra-oblast optimization reduces DALYs from 246,000 to 182,000, a reduction of 26%, while geographical optimization reduces it to 174,000, a further reduction of 4.4%. The relatively large improvement associated with intra-oblast optimization in the absence of constraints is due to the high effectiveness of the NSP intervention and its relatively low current funding levels, while the relatively small subsequent improvement from inter-oblast optimization is due to (a) funding already being well allocated between the three regions, (b) the similarity of the epidemic in each oblast and, as a consequence, (c) the interchangeability between oblasts of putting additional people on treatment (or enrolling them in the NSP).

## Discussion

Geographical targeting of HIV responses has become a dominant theme in the discussions and strategic thinking of both national and international health and development agencies. Both UNAIDS and the World Health Organization have increasingly emphasized the need to understand epidemic hotspots when designing HIV responses [16]. Two of the largest funders of the HIV response – the President’s Emergency Plan for AIDS Relief (PEPFAR) and the Global Fund to Fight AIDS, Tuberculosis and Malaria – have both pledged to focus efforts on pinpointing the geographic areas at sub-national levels with the highest disease burden in every country, so as to maximize resources and reach epidemic control [43, 44]. These efforts are also echoed in the national strategic plans of governments [45–47].

As a result, there is growing pressure to develop more geographically intricate models to guide decisions about how to allocate funding for HIV responses to the regions where it will have the greatest impact. The methods for geographical analysis presented here can be used to support the push towards further granularity in epidemic targeting [48]. Without this increased resolution, important inhomogeneities in socioeconomic status [49], cultural attitudes, rural-urban settings, and many other factors can be lost in the analysis, potentially leading to inaccuracy in models and subsequent policy advice.

An important use case for these methods is in guiding the allocation of funds across countries, thus enabling allocative efficiency to be conducted on a multi-country level rather than just an intra-country level. Such an analysis may be useful for international funding organizations that determine resource allocation policies not just in isolated countries but across entire regions. A recent study by Kelly et al. [20] illustrates that large potential gains – up to 1.9 million (33%) additional infections averted with no additional funding – might come from such a redistribution.

The geographical optimization method described here is one of many possible implementations; others have been investigated elsewhere [11]. Indeed, with the computational costs of applying the algorithm in full, it is an active area of research to seek alternate heuristic methods that trade accuracy for speed [12]. Even within the Optima suite of models, the geographical analysis algorithm has been fine-tuned for individual applications such as malaria [21] and nutrition [50]. However, all variants of geographical analysis implemented across the Optima suite share a core approach: a total budget is distributed across multiple independent regions by using a set of budget-outcome curves. Because budget-outcome curves are agnostic to the quantity being varied, they need not only be applied to allocating between different regions; they could even be used to allocate funding between different disease areas (e.g., one could define a diabetes BOC and a cardiovascular disease BOC, and optimally allocate funding between the two accordingly).

There are several limitations to the methodology presented here. First, as with all modeling, the quality of the outputs will be limited by the quality of the inputs, which varies considerably between countries and regions. This issue is especially pronounced for geospatial analyses, however, due to their much larger requirements for data. Although there is uncertainty in the inputs used in this model, we have not performed sensitivity or uncertainty analyses, which are important for ensuring the reliability of modeling results when used to inform real-world decision-making. Second, the approach of defining independent BOCs for each region assumes there is no interaction between regions. Although a reasonable assumption for medium- and large-scale regions (such as provinces or countries), this assumption becomes problematic if regions are defined at a fine resolution (for example, a square-kilometer grid). Finally, although the algorithm described here has been used at scale – including for 44 countries in [20], and for nearly 100 subnational districts for individual country studies – it has not yet been used for geographical analyses with thousands or hundreds of thousands of regions [48]. Although the algorithm is computationally efficient in that its computation time scales linearly with the number of regions, since it is nonetheless computationally intensive for each region, there is a practical upper limit on how many regions it can be applied to (approximately 50 to 500 for a typical laptop, or 1,000 to 10,000 for a typical high-performance computer). Thus, to optimize a large number of regions, additional steps – such as grouping regions hierarchically, and performing geographical optimizations at successively granular levels – are likely to be required. The level of detail required for an informative geographical analysis is determined by the spatial heterogeneity of the disease or condition being modeled, which in turn is determined largely by its latency and its dependence on geographical factors. For example, cholera and malaria, which have relatively short latencies and are highly dependent on geographical factors (such as access to clean drinking water and vegetation coverage), are likely to require a finer geographical scale than conditions such as HIV and tuberculosis, which have longer latencies and more dependence on social rather than geographical factors.

## Conclusions

This study demonstrates a method for determining geographical optimization of limited resources, illustrated with a case study of Ukraine. Critically, the methods applied here, especially the concept of the budget-outcome curve, are completely general and can be applied beyond geographical optimization to any problem where competing resources need to be allocated with constraints. Future applications will use this methodology to determine optimal resource allocations at higher spatial resolutions, and to determine tradeoffs in resource allocations between HIV, tuberculosis, and malaria.

## Supplementary information

**Additional file 1** Data and calibration. The data used, calibration, and incremental cost-effectiveness ratios for each oblast are provided in Appendix.pdf.

## Data Availability

The geographical analysis method is part of the Optima HIV tool, and is freely available on GitHub (http://github.com/optimamodel/optima). Code used to generate the figures used for this paper and the data used for the computational analysis are available upon request. Data from the Ukraine study are available contingent on approval from national stakeholders. All requests should be made via the corresponding author (Cliff Kerr, cliff@optimamodel.com).

## Abbreviations

AE: Allocative efficiency
AIDS: Acquired immune deficiency syndrome
ART: Antiretroviral therapy
BOC: Budget-outcome curve
DHS: Demographic and Health Surveys FSW: Female sex workers
HCT: HIV counseling and testing
HIV: Human immunodeficiency virus
ICER: Incremental cost-effectiveness ratio
MSM: Men who have sex with men
NSP: Needle-syringe program
OST: Opiate substitution therapy
PCHIP: Piecewise cubic Hermite interpolating polynomial
PEPFAR: U.S. President’s Emergency Plan for AIDS Relief
PWID: People who inject drugs
UNAIDS: Joint United Nations Programme on HIV/AIDS
USAID: United States Agency for International Development
USD: United States Dollar
