# Supplementary material for: Optimal allocation of HIV resources among geographical regions
Source: BMC Public Health. 2019 Nov 12;19:1509. doi: 10.1186/s12889-019-7681-5 (PMC6849208; doi:10.1186/s12889-019-7681-5)
Supplement: Supplementary file 1 — Additional file 1 Data and calibration. The data used, calibration, and incremental cost-effectiveness ratios for each oblast are provided in Appendix.pdf. [file 12889_2019_7681_MOESM1_ESM.pdf]

Appendix A

Program costing data collection was conducted by the Ukrainian Institute on Public Health Policy and took place between May and July 2016. Staff visited each facility to collect aggregate level facility data. Data sheets contained information regarding both direct costs (traceable to patient services) and indirect (overhead) costs, as well as programmatic data related to the numbers of clients receiving HIV services. All cost data were collected in Ukrainian Hryvnia (UAH) but converted to United States Dollars (USD) based on the 2015 average exchange rate of 21.8447 UAH to 1 USD. Cost data were gathered according to the ingredient approach, meaning that resource quantities, and their respective costs, were collected separately. Both direct and indirect costs were self-reported to data collectors during in-person site visits. All inputs were valued at their opportunity costs determined by market prices. Program data are shown in Table A1.

Epidemiological data were collected for 11 population groups in each oblast: female sex workers (FSW), clients of sex workers, men who have sex with men (MSM), people who inject drugs (PWID), prisoners, and general population males and females aged 0–14, 15–49, and 50+. Data were based on all available sources, including Spectrum data, a previous Optima HIV study (World Bank and UNAIDS, 2015: *Value for Money in Ukraine's HIV response: Strategic investment and improved efficiency*), and the National Public Health Center. All data were reviewed by in-country experts. Model calibrations to the epidemiological data for each oblast are shown in Figures A1-A6 below.

Incremental cost-effectiveness ratios (ICERs) for each program and each oblast are shown in Figures A7-A9 relative to baseline spending. However, note that these ICERs are specific to the epidemic context at a given point in time and on the coverage values of the other programs; the values of the ICERs would differ if they were calculated relative to the optimized budget, for example.

Table A1 Program data used in the case study.

Program	Oblast	Unit cost	Coverage	Total cost	Impact of being covered
ART	Mykolayiv	$693.46	4,498	$3,041,531.80	Person is on ART
	Poltava	$748.41	1,735	$1,214,668.20	
	Zhytomyr	$671.86	1,746	$773,312.85	
HCT	Mykolayiv	$6.01	89,495	$398,169.08	General population: test every 2 years; key population: test every 6 months
	Poltava	$14.71	17,227	$215,281.53	
	Zhytomyr	$6.06	41,905	$152,384.58	
Condoms	Mykolayiv	$2.20	6,576	$14,467.20	Condom use in casual relationships increases from 50% to 80%
	Poltava	$2.20	15,044	$33,096.80	
	Zhytomyr	$2.20	1,063	$2,338.60	
NSP	Mykolayiv	$45.09	2,299	$103,666.34	Needle-syringe sharing rate decreases from 35% to 3%
	Poltava	$60.90	1,699	$103,453.14	
	Zhytomyr	$56.55	1,525	$86,206.80	
OST	Mykolayiv	$364.12	710	$256,703.49	Person is on OST (and stops injecting)
	Poltava	$486.00	584	$282,365.90	
	Zhytomyr	$249.56	340	$81,854.24	

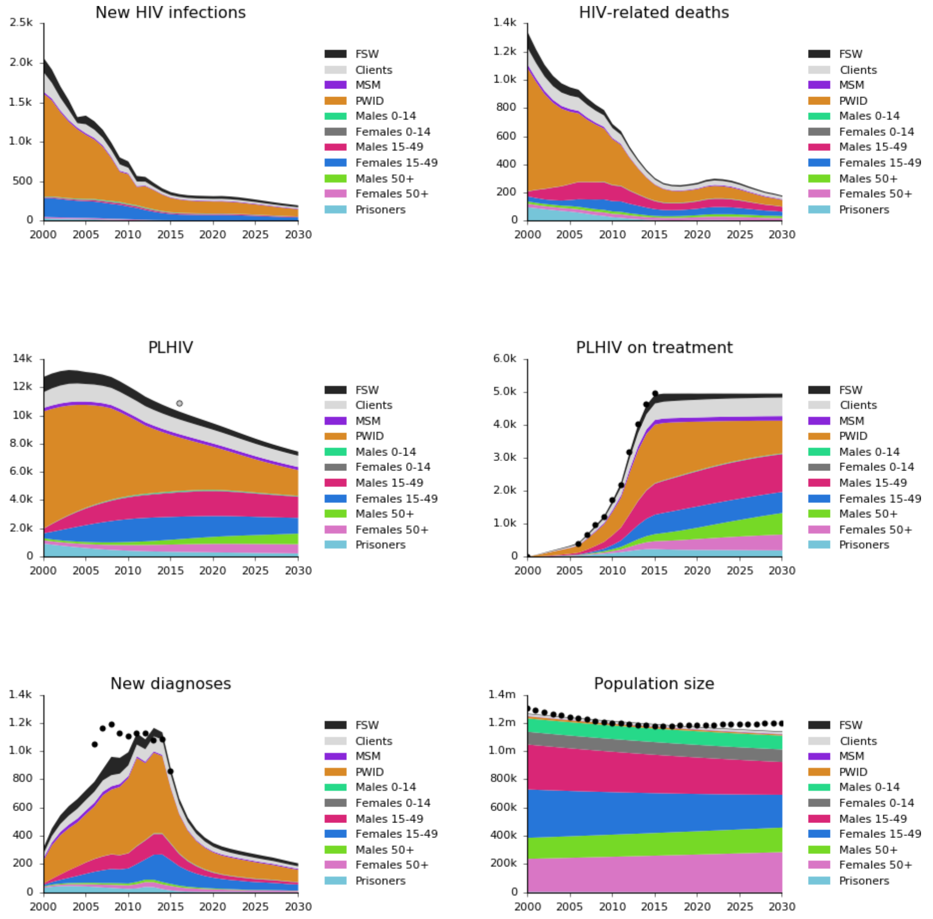

Figure A1 Calibration for Mykolayiv to data on new HIV infections, HIV-related deaths, care cascade, and other data.

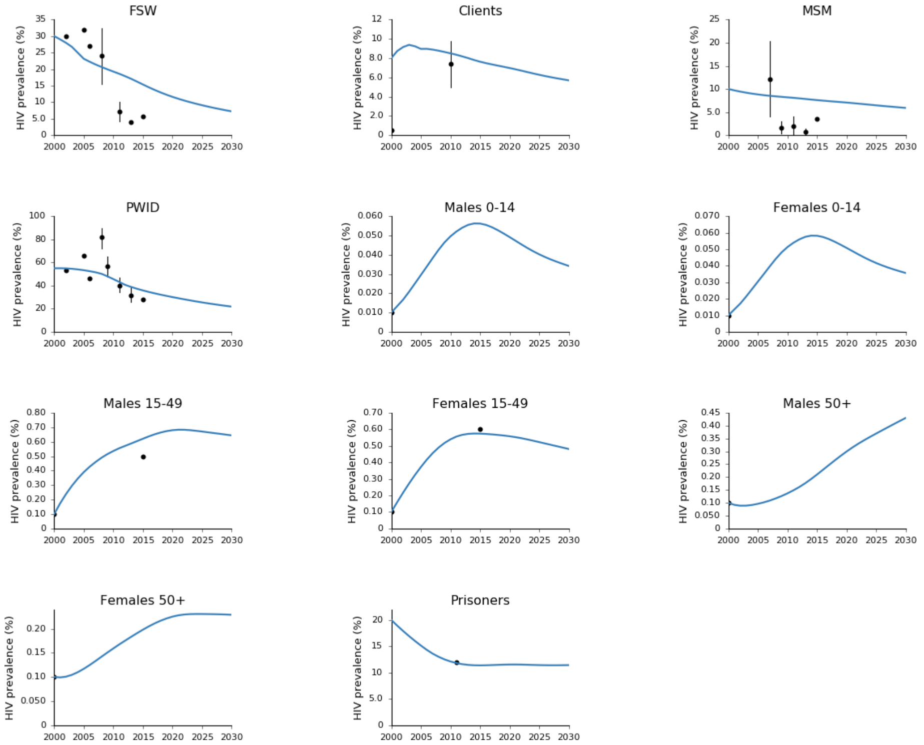

Figure A2 Calibration for Mykolayiv to data on HIV prevalence by population.

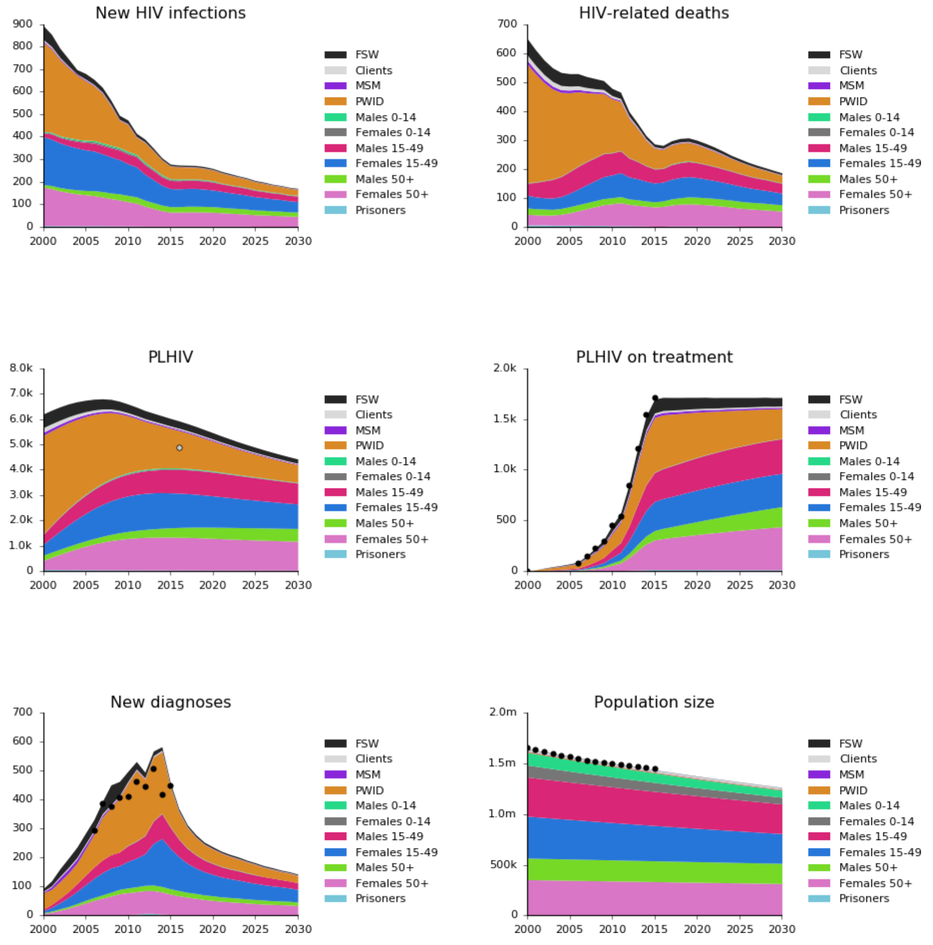

Figure A3 Calibration for Poltava to data on new HIV infections, HIV-related deaths, care cascade, and other data.

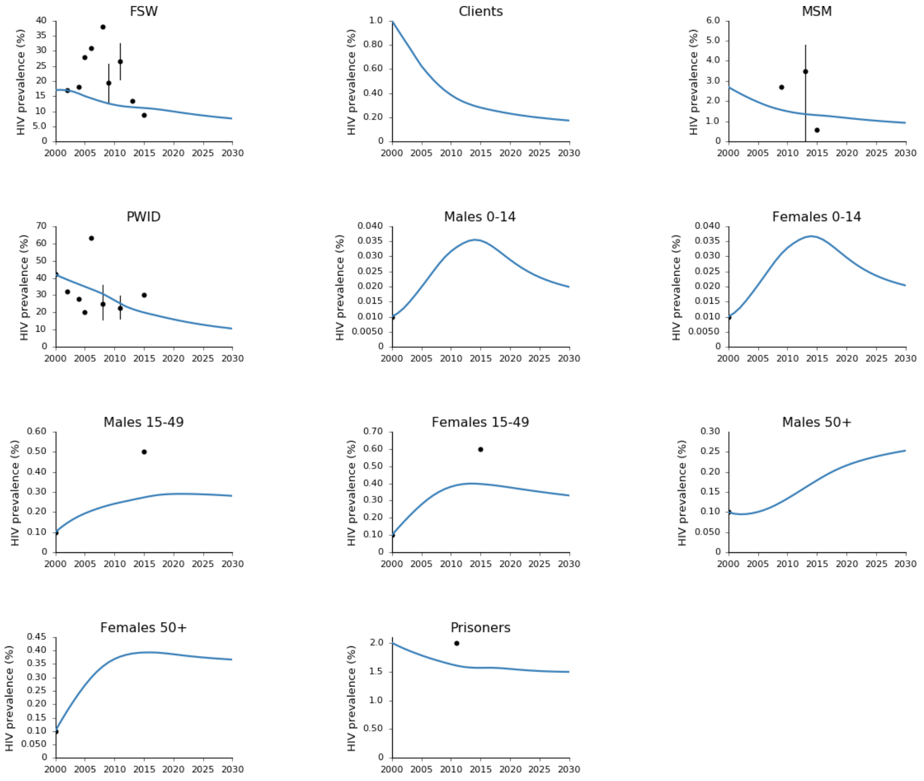

Figure A4 Calibration for Poltava to data on HIV prevalence by population.

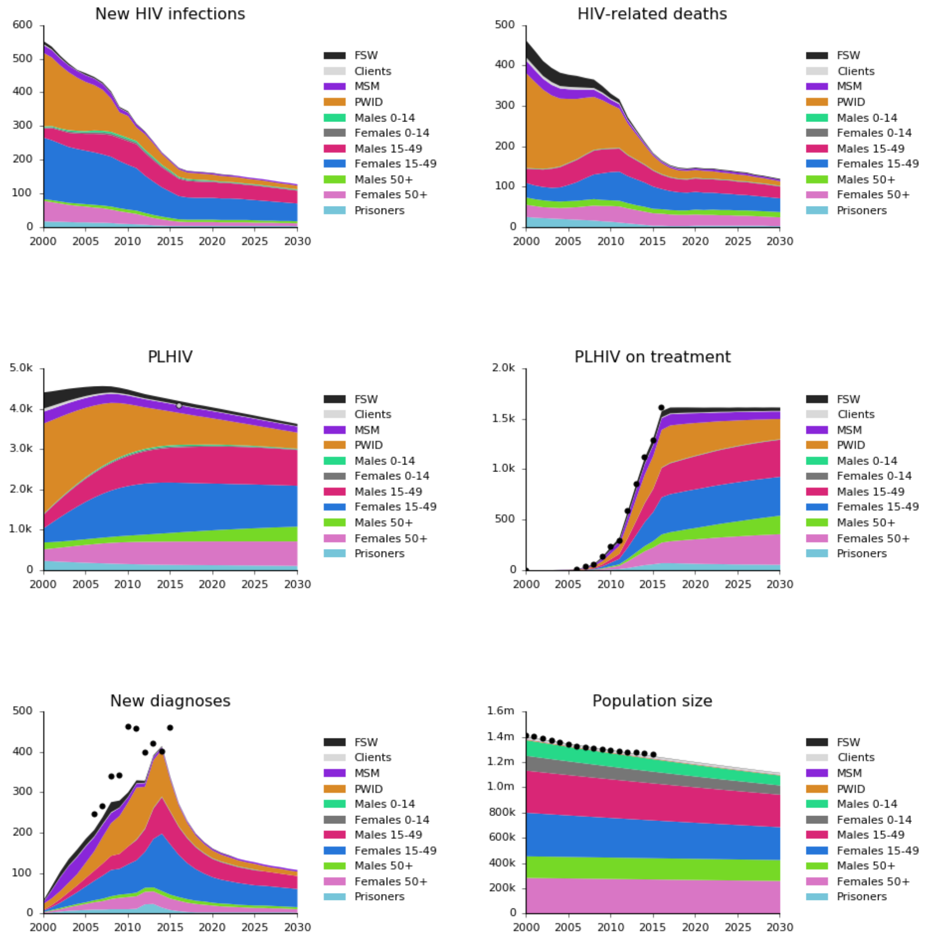

Figure A5 Calibration for Zhytomyr to data on new HIV infections, HIV-related deaths, care cascade, and other data.

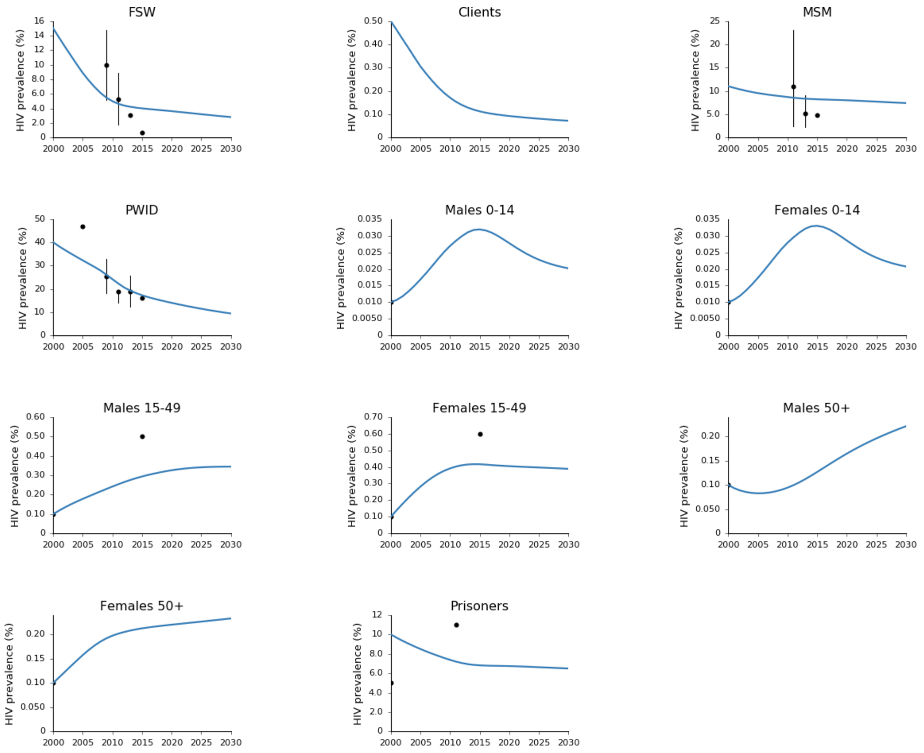

Figure A6 Calibration for Zhytomyr to data on HIV prevalence by population.

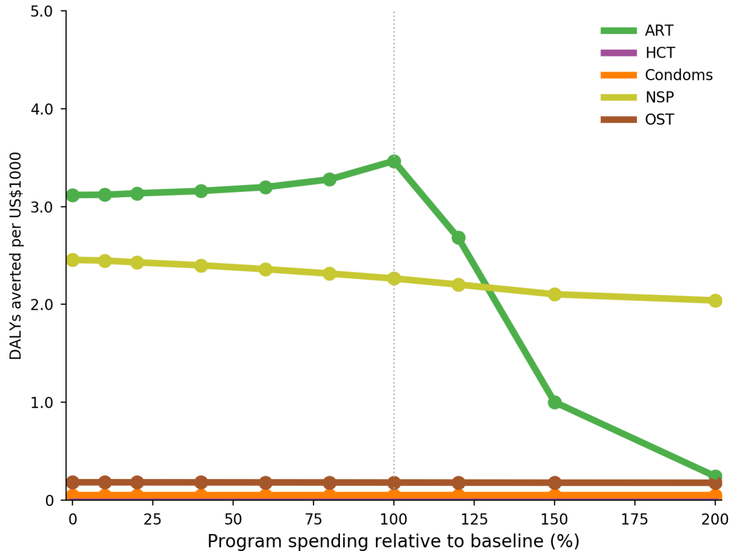

Figure A7 Inverse incremental cost-effectiveness ratios (ICERs) for each program in Mykolayiv relative to baseline spending. The reciprocals of the ICERs are shown so that programs with higher cost-effectiveness have larger values.

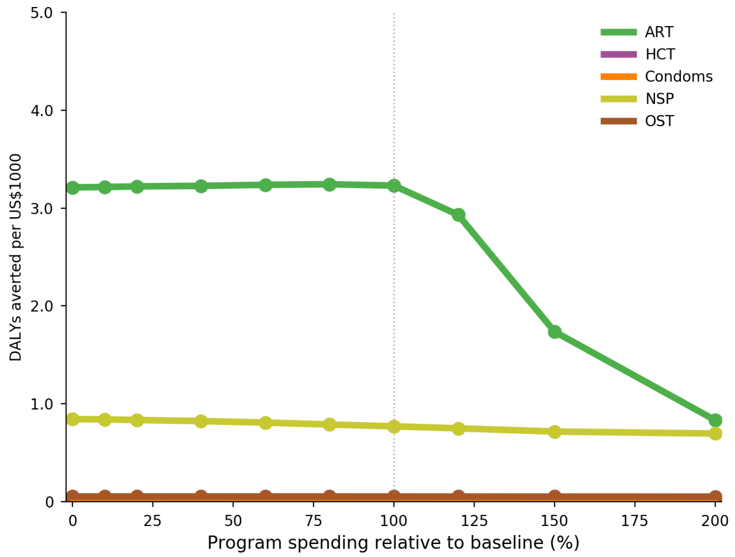

Figure A8 Inverse incremental cost-effectiveness ratios for each program in Poltava relative to baseline spending.

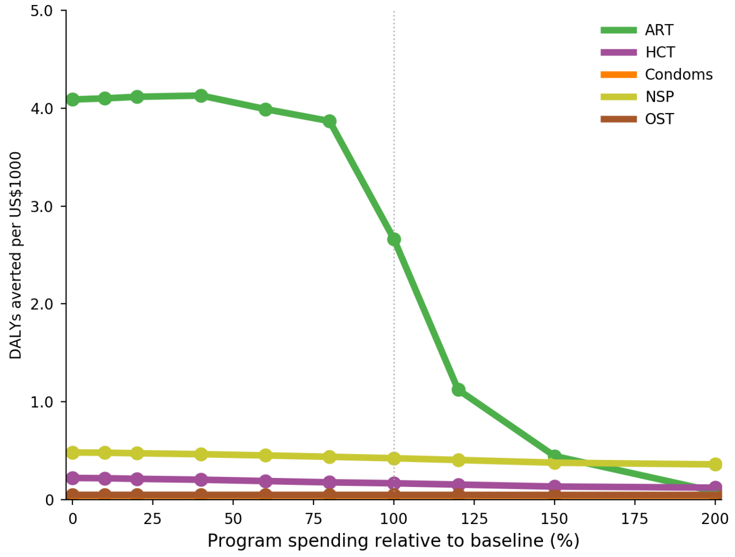

Figure A9 Inverse incremental cost-effectiveness ratios for each program in Zhytomyr relative to baseline spending.
